# Artificial Life Approach for Controlling Fibrillation in Self-Oscillating Gels

**DOI:** 10.1021/acsomega.6c03790

**Published:** 2026-07-24

**Authors:** Shun Oi, Ryuhei Sato, Ryo Yoshida, Yasushi Shibuta

**Affiliations:** Department of Materials Engineering, 13143 The University of Tokyo, 7-3-1 Hongo, Bunkyo-ku, Tokyo 113-8656, Japan

## Abstract

Biological rhythms are fundamental to life but are highly complex and difficult to study directly in living systems. Understanding how these rhythms can be controlled is crucial for addressing rhythm disorders. This challenge has increased interest in software-based Artificial Life (ALife) approaches, which allow systematic investigation of rhythmic phenomena under controlled conditions. Here, we numerically examined the response of self-oscillating gels to external mechanical forces, focusing on fibrillation control and resonance. A coupled model of the Belousov–Zhabotinsky (BZ) reaction and gel volume changes reproduced the gel’s intrinsic oscillations. Fibrillation, characterized by stable spiral waves and suppressed swelling/contraction, was induced by inclined compression during early swelling. Applying multiple compressions at intervals corresponding to integer ratios of the gel’s intrinsic period suppressed fibrillation, demonstrating resonance. The gel’s oscillation period also synchronized with compressions near its intrinsic period or harmonics. These results highlight the potential of ALife approaches to elucidate mechanisms of biological rhythm disorders and suggest strategies for restoring coordinated oscillations, analogous to the resuscitation of a heart in a fibrillatory state.

## Graphical abstract

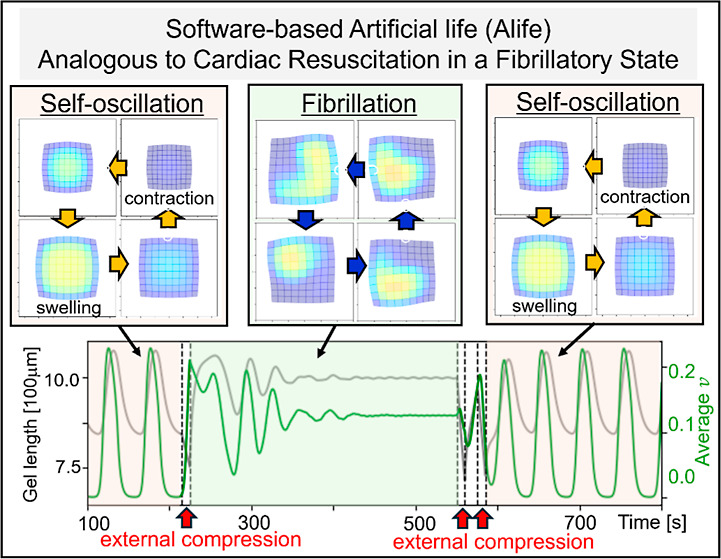

## 1 Introduction

The understanding of temporally varying dynamic behaviors exhibited by living organisms, such as heartbeat, respiration, neural activity, and metabolism, is considered crucial for assessing health conditions and diagnosing abnormalities, and even developing artificial life systems.^1,2^ However, direct observation of these biological rhythms is limited; for example, while heartbeats can be analyzed using electrocardiography, signal acquisition through the skin is subject to significant noise and attenuation. Therefore, A new approach called artificial life (ALife),^3^ which seeks to understand the essence of life by constructing artificial systems based on hypotheses about its fundamental nature, has attracted considerable attention. The principal methodologies in Alife comprise software-based approaches utilizing computers, hardware-based approaches employing physical machines such as robots, and wet approaches grounded in chemistry.^4^ In this context, self-oscillating gels we developed,^5–7^ which convert chemical energy into mechanical energy in the form of swelling and contraction, have attracted considerable attention. Various biomimetic actuators composed of self-oscillating gels have been presented including self-walking,^8^ self-propelled motion,^9^ and periodic reciprocating motion.^10^ These gels are a type of soft material that artificially reproduces oscillatory phenomena characteristic of life by generating an internal oscillatory reaction known as the Belousov–Zhabotinsky (BZ) reaction.^11,12^ The BZ reaction is a nonequilibrium chemical reaction, sharing with biological metabolism the common feature of existing in a nonequilibrium state. Its periodic oscillations closely resemble biological rhythms such as heartbeat and respiration, making it promising for applications in physiological function research.^13,14^ Furthermore, these periodic oscillations are also analogous to biological growth processes occurring under time-varying external fields, such as fluctuating light or temperature conditions.^15^

To elucidate the oscillatory behavior of the BZ reaction from a theoretical perspective, the FKN mechanism was proposed.^16^ This mechanism, although comprising ten elementary reactions, fundamentally relies on the alternation between two pathways of bromine production, which gives rise to oscillations. From the FKN framework, the essential reaction steps were extracted to formulate a simplified mathematical representation of the BZ reaction, known as the Oregonator.^17^ In particular, the Keener–Tyson variant of the Oregonator model^18^ is expressed in terms of two variables: an activator (*u*, proportional to the concentration of HBrO_2_) and an inhibitor (*v*, proportional to the concentration of Ce^4+^). Despite its simplicity, it has been shown to reproduce the oscillatory behavior of the BZ reaction as well as spatiotemporal patterns such as target patterns and spiral waves. For example, unidirectional propagation of BZ reaction in triangle and pentagonal arrays of self-oscillating gel was investigated using the Keener-Tyson model.^19^ Furthermore, a model coupling the BZ reaction with the volume changes of the gel have been proposed,^20,21^ enabling the reproduction of the periodic oscillations of self-oscillating gels through numerical simulation.^20–23^

Recently, it has been reported that externally applied periodic mechanical stimulations induce synchronization of chemical oscillations within the gel, leading to a resonance phenomenon in which the reaction cycle of the gel under compression and the compression cycle exhibit an integer ratio.^24^ These results demonstrate that the self-oscillating gel is capable of responding to external stimuli, implying potential applications in the regulation of gel behavior via mechanical stimulation. Based on this background, the present study aims to understand how self-oscillating gels respond to external mechanical forces through numerical simulations. In particular, this study conducts simulations of fibrillation mimicking atrial fibrillation, focusing on its manifestation and suppression. Atrial fibrillation in the ventricle is known to manifest as a spiral wave,^25,26^ which is analogous to the spiral wave dynamics exhibited by the BZ reaction. This study demonstrates the significance of Alife by examining resuscitation from a fibrillation state through external mechanical stimulation in phenomena, particularly for phenomena that are largely inaccessible to experimental investigation in living organisms.

## 2 Simulation Methodology

The mathematical model of the self-oscillating gel employed in this study is essentially based on the work of Yashin et al.^21^ and is formulated as a simplified two-dimensional model to facilitate implementation. In overview, reproducing the behavior of self-oscillating gels requires coupling a model that describes the internal BZ reaction with a model that accounts for the structural changes of the gel. A detailed description of the mathematical model for the self-oscillating gel is provided in Section S1 of the Supporting Information. A modified Keener-Tyson model is employed to describe the dynamic concentration changes of the BZ reaction within the gel. The dimensionless concentrations of the activator *u* (proportional to HBrO_2_ concentration) and the inhibitor *v* (proportional to Ce^4+^ concentration) are described by the following equations

1 $$\frac{\partial u}{\partial t}=-\nabla \cdot j\left(u\right)-\nabla \cdot \left(uv^{\left(s\right)}\right)+F\left(u,v,\phi \right)$$

2 $$\frac{\partial v}{\partial t}=-\nabla \cdot \left(vv^{\left(p\right)}\right)+\varepsilon G\left(u,v,\phi \right)$$

3 $$F\left(u,v,\phi \right)={\left(1-\phi \right)}^{2}u-u^{2},-\left(1-\phi \right)fv\frac{u-q{\left(1-\phi \right)}^{2}}{u+q{\left(1-\phi \right)}^{2}}$$

4 $$G\left(u,v,\phi \right)={\left(1-\phi \right)}^{2}u-\left(1-\phi \right)v$$

here, ***j*** (*u*) denotes the diffusion flux, which is given by eq S9 in the Supporting Information. ***v*** ^(p)^ and ***v*** ^(s)^ represent the velocities of the polymer and the solvent, respectively. ε is the ratio of reaction rates, and *q* and *f* are reaction parameters. ϕ is the polymer volume fraction. The continuity equation for the polymer volume fraction is expressed as

5 $$\frac{\partial \phi }{\partial t}=-\nabla \cdot \left(\phi v^{\left(p\right)}\right)$$

A gel lattice spring model is adopted to describe the flexibility of the gel following the work of Yashin et al.^21^ This model discretizes the object into lattice points, with each point connected by springs. The motion of each lattice point *m* = (*k*,*l*) is described as an overdamped oscillation without inertia

6 $$\frac{\partial x_{m}}{\partial t}=M_{m}F_{m}$$

here, *M*_*m*_ is called the mobility, indicating that the velocity of the gel lattice point is proportional to the force acting on that part. The force ***F***_*m*_ acting on each lattice point *m* = (*k*,*l*) is determined by the elastic forces exerted by neighboring lattice points and the osmotic pressure force, as defined by eq S14 in the Supporting Information.

The models described above are implemented according to the following procedure, and numerical solutions are obtained through computational simulation. First, in accordance with eq 6, forces arising from elasticity and osmotic pressure are applied to the nodes, and their coordinates are updated. Next, the polymer velocity ***v*** ^(p)^ is obtained from the nodal motion, the solvent velocity ***v*** ^(s)^, and the polymer volume fraction ϕ from the volume of each element. These values are subsequently used to update the reactant concentrations, *u* and *v* in eqs 1 and 2. Repeating this sequence yields the simulation of the self-oscillating gel model. For the numerical simulations, the partial differential equations were discretized using the finite difference method. The Laplacian was approximated by the second-order centered difference scheme, while the time derivative was treated using the first-order forward difference scheme. This approach is a well-established solution method for governing equations based on diffusion processes.^27^ The calculation cell for a two-dimensional square-shaped gel is discretized into 10 × 10 grid of lattice points, except for the comparison with experiments in Figure S2, where a 20 × 20 grid is used for verification. The initial grid spacing was defined as 5 × 10^–5^m. It should be noted that the grid spacing was allowed to vary during the calculation in accordance with the volumetric expansion or contraction of the gel. Each grid point is assigned the variables *u*, *v*, and ϕ. The parameters employed in this study follow those reported by Yashin et al.^21^ The parameters for the BZ reaction are *q* = 9.52 × 10^5^, ε = 0.354, and *f* = 0.7. Gel structural parameters include χ_0_(25^◦^ *C*) = 0.338 and χ_1_ = 0.518 for the osmotic pressure-related parameter χ­(ϕ). The spring constant is *K* = 1.3 × 10^–3^, the diffusion coefficient *D*_u_ = 2 × 10^–5^cm^2^s^–1^, and the friction coefficient *A*_0_ = 100. The activator concentration, *u* outside the gel is fixed at 0, assuming rapid decomposition. The computational time step is set to Δ*t* = 1.0 × 10^–3^s. A handmaid code written by Python is used for numerical simulations. All computations are performed on a workstation with Intel­(R) Xeon­(R) w7-3455 CPU (2.60 GHz).

Prior to conducting the simulations central to this study, the self-oscillating gel model was validated using a square-shaped gel. Further details are provided in Section S2 of the Supporting Information. The model was found to reproduce self-oscillation and accurately capture the intrinsic oscillatory and structural dynamics of the self-oscillating gel (Figure S1 in the Supporting Information). Moreover, comparison with experimental observations^28^ demonstrated that the simulated patterns are in good agreement with those observed experimentally, both during the initial stage of the reaction and in the subsequent stable phase (Figure S2 in the Supporting Information). Further details are provided in Section S2 of the Supporting Information.

## 3 Results and Discussion

### 3.1 Manifestation of Fibrillation in a Self-Oscillating Gel

In this study, fibrillation was artificially induced by modifying the internal reaction pattern through external compression. Our preliminary investigations indicated that inclined compression of square gels during the early swelling phase induced fibrillation, and thus these conditions were adopted in the present study. Compression was imposed at an inclined angle from 215 to 225 s. The left side of the gel was fixed, while the upper and lower ends were compressed down to 60% and 80% of the swollen-state length (1070 μm), respectively. The subsequent compression ratio was similarly defined with reference to the swollen-state length to ensure a unified scale for discussion. Note that the absolute displacement may vary depending on the initial state of the gel at the onset of compression. Figure 1a shows the temporal variation of the gel length along the left–right direction and the spatially averaged *v* during the swelling–contraction cycles, where a 10 s compression was applied and subsequently led to fibrillation. The interval between the dotted lines in the figure corresponds to the 10 s period during which the gel is subjected to compression. Examination of the averaged *v* before and after compression reveals a small reaction immediately following compression; however, the peak amplitude gradually decreases and eventually stabilizes. Correspondingly, the change in gel length also approaches a nearly constant value, resulting in a fibrillated state in which further swelling and contraction are suppressed. Figure 1b,c show snapshots of the process of fibrillation development and the state of the gel after its occurrence. Detailed snapshots over an extended period are provided in Figure S3 and the corresponding dynamics are provided in Movie S2 in Supporting Information. Immediately after compression, the entire gel exhibited a reaction, with activity first appearing in the lower right region, which experienced less compression, and subsequently in the upper right region, which was subjected to greater compression. This resulted in vortex-like propagation of the reaction as shown in Figure 1c. Spiral waves, a characteristic feature of the BZ reaction, were observed in the fibrillated gel. The rotation period of the spiral wave was 27.7 s. This is shorter than the period of self-oscillation prior to compression, which was 51.1 s. This reduction in period is consistent with the characteristic behavior of spiral waves in the conventional BZ reaction.

**Figure 1:**
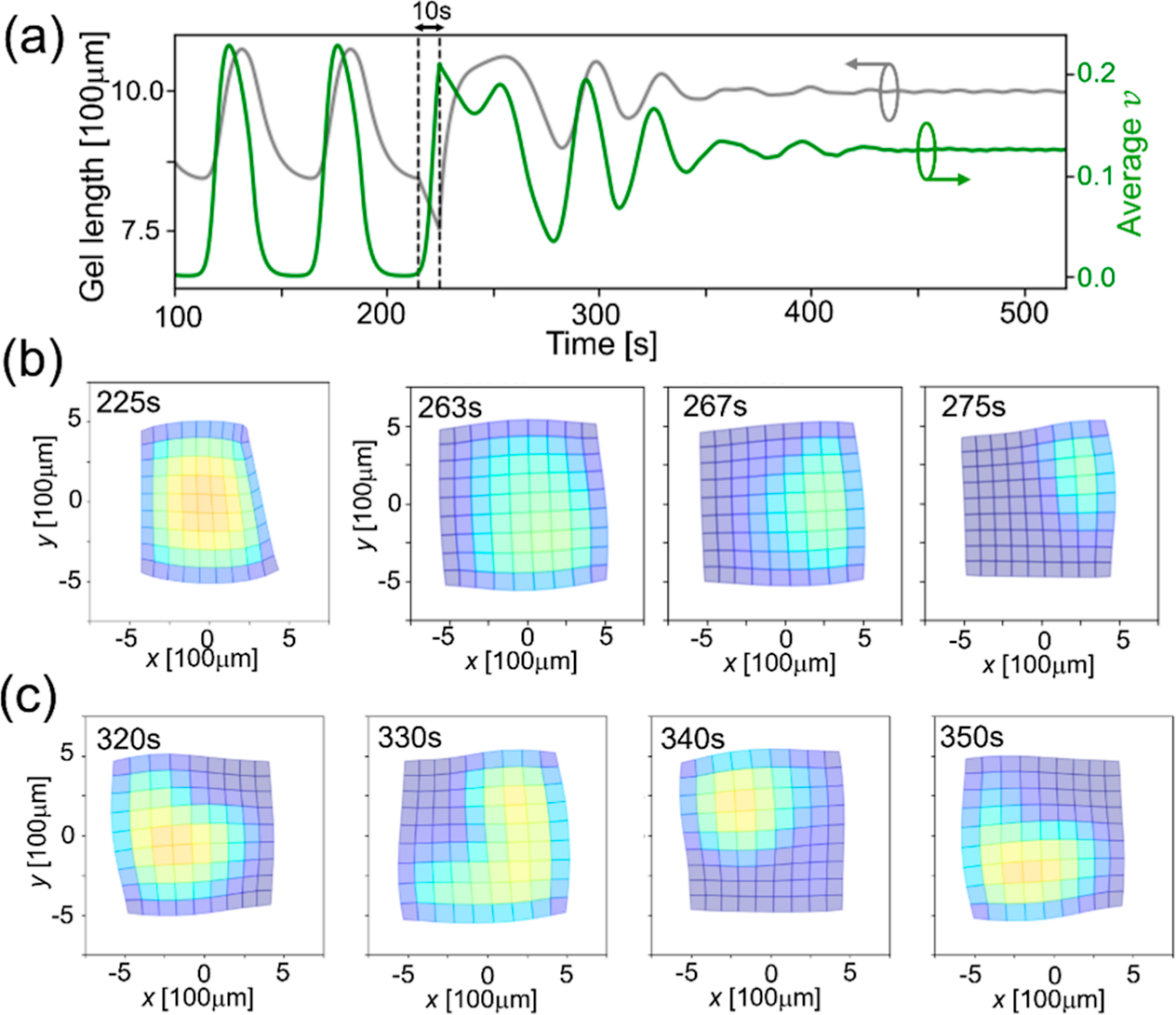
(a) The temporal variation of the gel length along the left–right direction (gray curve) and the spatially averaged *v* (green curve) during the swelling–contraction cycles, where a 10 s compression was applied and subsequently led to fibrillation. The arrows identify the corresponding axis for each curve. The same color convention is adopted throughout the subsequent figures. The interval between the dotted lines corresponds to the 10 s period during which the gel is subjected to compression. Snapshots of (b) the process of fibrillation development and (c) the state of the gel after its occurrence. Colors indicate the concentration distribution of *v*.

Interestingly, when the compression gradient was reversed (80% and 60% on the upper and lower sides, respectively), the direction of the spiral wave was also reversed. Details are provided in Figure S4 in Supporting Information. In Figure 1c, counterclockwise spiral waves were observed, whereas clockwise spiral waves were observed in the case with reversed gradient. This reversal arises because, immediately after compression, waves propagate from the less-compressed upper right region toward the more-compressed lower right region. On the other hand, it was confirmed that fibrillation did not occur when the compression gradient was small. Specifically, no fibrillation was observed when the upper end was compressed down to 70% and the lower end down to 75%. Immediately after compression, partial reactions similar to those in the previous cases were observed. These peaks then disappeared, and the reaction propagated throughout the gel from the upper left region. Details are provided in Figure S5 in Supporting Information. Furthermore, no fibrillation was observed when compression was applied during different phases of oscillation in the gel. An inclined compression with the same gradient as in Figure 1 (60% at the upper end and 80% at the lower end) was applied at three different stages: early contraction, late contraction, and late swelling. Fibrillation was not observed even under the same inclined compression, indicating that it occurs only when compression is applied during a specific phase of the early swelling period. The effect of compression timing on the response of the self-oscillating gel is further discussed in the Discussion section.

### 3.2 Suppression of Gel Fibrillation via Additional Compression

Subsequently, the effect of applying additional external compression to return the gel to a self-oscillating state after fibrillation onset was examined. As the first case, the gel was compressed once from 550 to 560 s down to 70% of its swollen-state length. Figure 2a shows the temporal variations in the left–right gel length and the spatially averaged *v*. Consequently, fibrillation could not be completely eliminated. Examination of the snapshot in Figure 2b demonstrated that spiral waves expanded over a larger area immediately following compression, and the corresponding graph showed a strong emergence of oscillatory activity immediately thereafter. However, over time, these oscillations diminished and fully developed spiral waves reemerged. The corresponding dynamics are provided in Movie S3 in Supporting Information. A similar investigation was conducted with the compression ratios changed down to 75% and 80% of the length in the swollen state; however, fibrillation could not be eliminated in either case (see Figure S6 in the Supporting Information).

**Figure 2:**
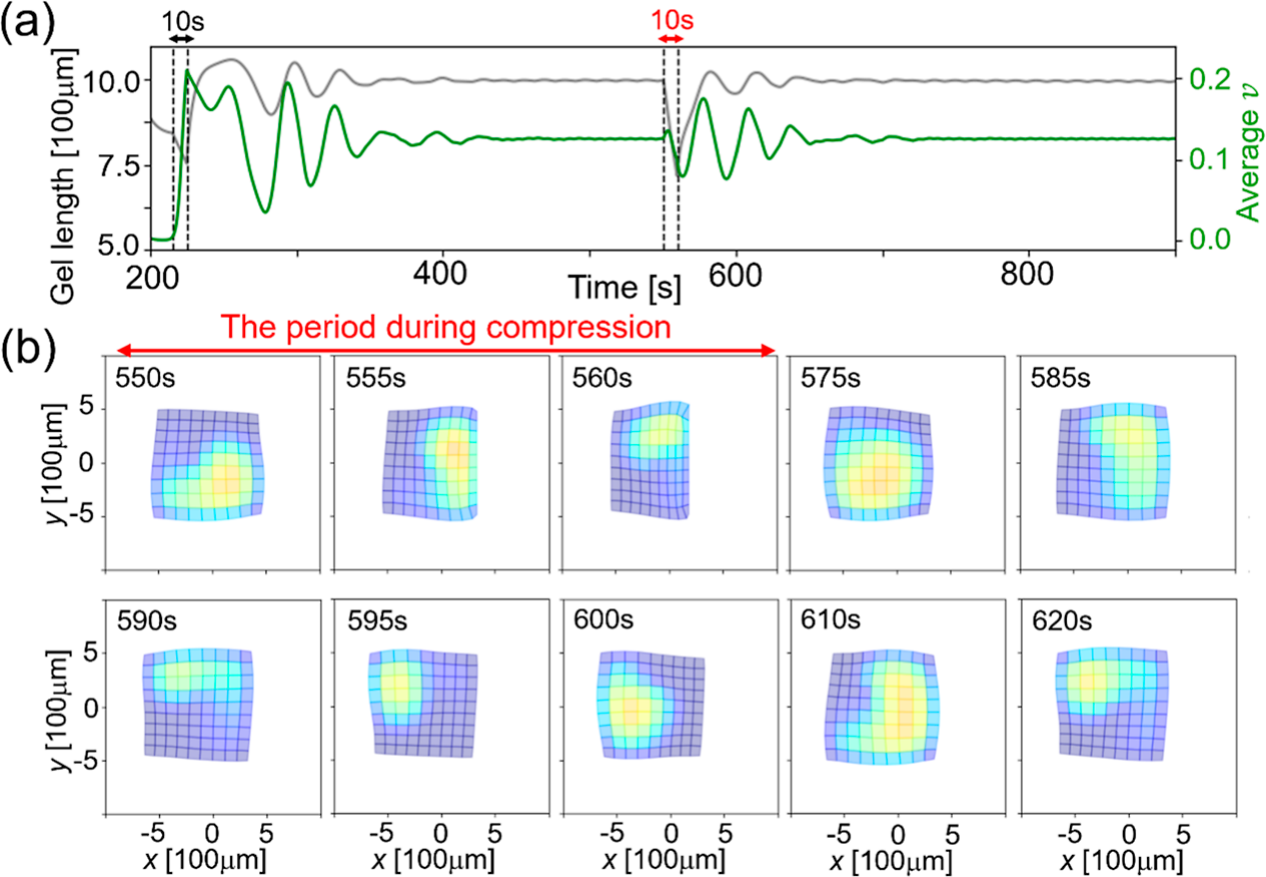
(a) The temporal variation of the gel length along the left–right direction (gray curve) and the spatially averaged *v* (green curve) during applying a single additional compression down to 70% of its swollen-state length to the fibrillated gel at 550 s. The interval between the dotted lines corresponds to the 10 s period during which the gel is subjected to compression. (b) Snapshots of the gel after a single additional compression to the fibrillated gel. Colors indicate the concentration distribution of *v*.

**Figure 3:**
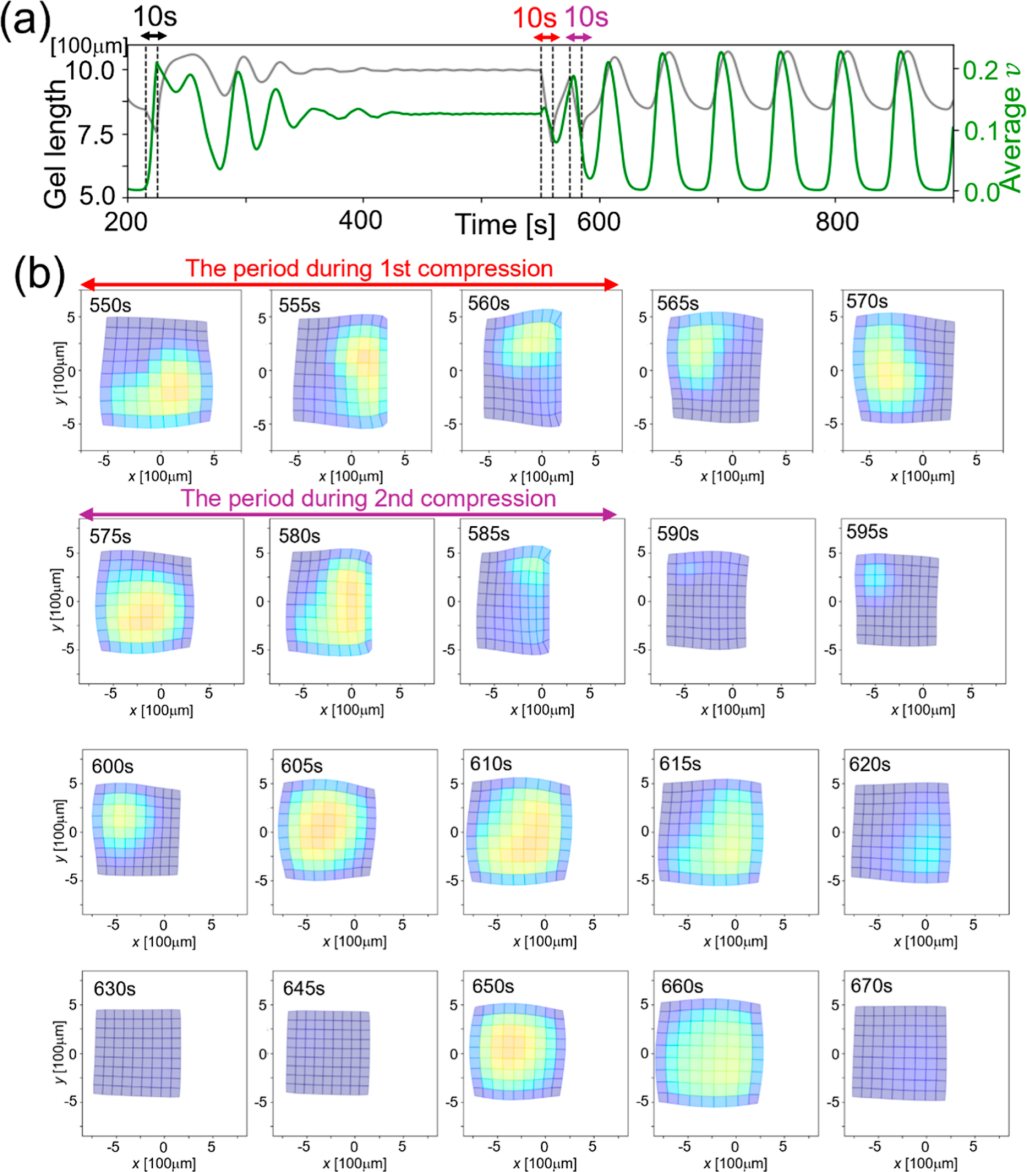
(a) The temporal variation of the gel length along the left–right direction (gray curve) and the spatially averaged *v* (green curve) during applying two additional compressions to the fibrillated gel. The interval between the dotted lines corresponds to the 10 s period during which the gel is subjected to compression. (b) Snapshots of the gel after two additional compressions to the fibrillated gel. Colors indicate the concentration distribution of *v*.

Subsequently, two compressions were imposed on the fibrillated state. The compression ratio was maintained at 70% of the swollen-state length, consistent with the previous case, and the compressions were applied at 550 and 575 s Figure 3 shows the temporal variation of the gel length along the left–right direction and the spatially averaged *v*, and snapshots of the gel during applying two compressions to the fibrillated gel. The corresponding dynamics are provided in Movie S4 in Supporting Information. Application of two compressions at 550 and 575 s successfully eliminated fibrillation, returning the gel to its swelling–contracting state. By approximately 590 s following the second compression, the peak associated with the spiral wave in the gel was no longer detectable. Thereafter, a reaction initiated in the upper left region, where activity had ceased, and propagated throughout the gel, resulting in a uniform reaction that restored the gel to its self-oscillating swelling–contracting state.

The effects of compression intensity and interval on the elimination of the fibrillated state were investigated. Figure 4a shows the temporal variation of the gel length in the left–right direction and the spatially averaged *v* during the application of two additional compressions at 80% of the swollen-state length, applied at the same interval. The gel emerged from the fibrillated state and transitioned to the swelling–contracting state, as observed in the case with a compression ratio of 70% of the length in the swollen state. The difference was that, for the 70% case, the gel exited the fibrillated state immediately after the two compressions, whereas for the 80% case, several tens of seconds were required for this transition. This delay indicates that the spiral waves were not completely eliminated immediately following compression.

**Figure 4:**
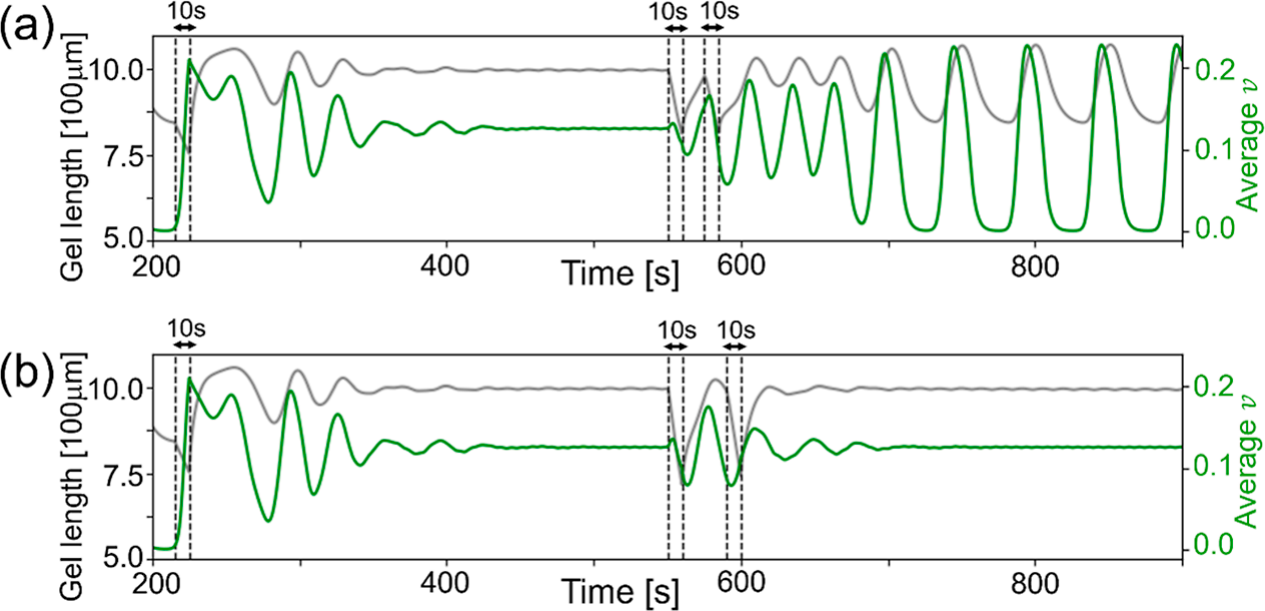
Temporal variation of the gel length along the left–right direction (gray curve) and the spatially averaged *v* (green curve) for (a) a compression ratio of 80% of its swollen-state length applied at 550 and 575 s, and (b) a compression ratio of 70% of its swollen-state length at 550 and 590 s. The interval between the dotted lines corresponds to the 10 s period during which the gel is subjected to compression.

Figure 4b presents the results for two compression cycles applied at 550 and 590 s, with a compression ratio of 70% of the initial size. Under these conditions, the gel remained in the fibrillated state even after the two compressions. These results indicate that, even at the same compression intensity, fibrillation may or may not be suppressed depending on the interval between multiple compressions. A possible explanation is that, in the former case where fibrillation was successfully suppressed, the interval between the two compressions was 25 s, which corresponds to approximately half of the intrinsic period of the self-oscillating gel (51.1 s) observed in Figure S1. In contrast, in the latter case, where fibrillation was not suppressed, the interval was 40 s, which deviates from both the intrinsic and harmonic periods of the gel. These results suggest that applying external forcing with a period equal to, or an integer multiple or fraction of, the intrinsic period of the self-oscillating gel can restore a fibrillated gel to its normal oscillatory state. This phenomenon, referred to as resonance, has been demonstrated experimentally: periodic compression of a self-oscillating gel induces synchronization of the reactions within the gel with the applied compression.^24^ Therefore, the following section investigates the effect of periodic external compression on the oscillation period of the self-oscillating gel.

### 3.3 Timing-Dependent Response of Self-Oscillating Gels to a Single Compression

To investigate the effect of compression timing on the response of self-oscillating gels, a gel exhibiting the intrinsic oscillation shown in Figure S1 was subjected to a single 65% compression at four different phases of the oscillation cycle: early contraction, late contraction, early swelling, and late swelling. To represent these phases, separate simulations were conducted with compressions applied during 185–195 s, 200–210 s, 215–225 s, and 225–235 s, respectively. Figure 5 shows the temporal variations of the gel length and averaged *v* in response to the compression applied at four different phases within the gel oscillation cycle. When the compression was applied during the early contraction phase, little change was observed following the compression. During the late contraction phase, compression induced a slight delay in the postcompression reaction phase. Compression applied at the early swelling phase resulted in an unstable response immediately after compression, followed by a substantial delay in the subsequent reaction phase. Finally, compression applied during the late swelling phase caused the postcompression reaction phase to advance. These results indicate that the timing of compression strongly influences the subsequent gel response. When the gel was compressed at phases other than early swelling, the oscillation exhibited either no noticeable phase shift or only a slight deviation. In contrast, compression applied during the early swelling phase resulted in an unstable response, leading to markedly different behavior. This outcome is fully consistent with physical expectations, as the forced compression applied at the onset of swelling compels the gel’s volume change to proceed in the opposite direction, thereby inevitably inducing unstable behavior. This finding provides direct evidence for the observation in Figure 1, namely that fibrillation occurred exclusively when an inclined compression was applied during the early swelling phase and did not occur at other phases. In other words, it clearly demonstrates that forced compression at the onset of swelling can artificially destabilize the gel’s response.

**Figure 5:**
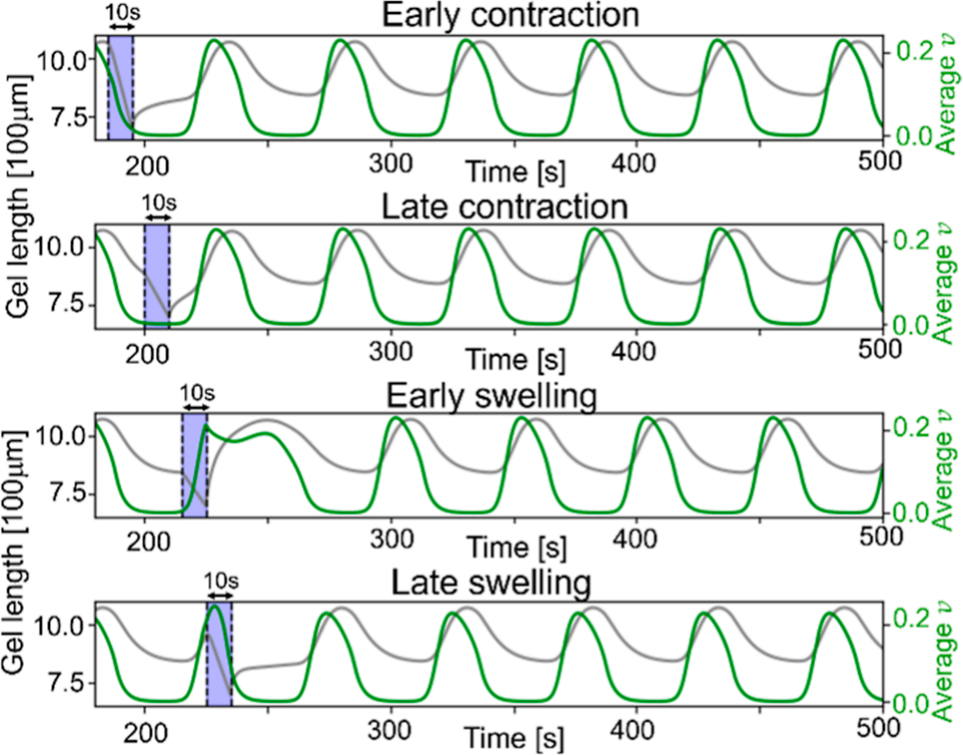
Temporal variations of the gel length along the left–right direction (gray curve) and the spatially averaged *v* (green curve) in response to a single 65% compression at four different phases within the gel oscillation cycle: early contraction, late contraction, early swelling, and late swelling. The gel was compressed during the 10 s intervals highlighted by the shaded regions enclosed by dashed lines.

### 3.4 Resonance of Gel Self-Oscillation under Periodic External Compression

Here, we investigated the resonance behavior of the self-oscillation period of the gel in response to external compressions applied with various periods. In addition to the conditions used in Figure S1, periodic compressions were applied to the self-oscillating gel from 500 to 1500 s. The gel was compressed to 65% of the swollen-state length at different periods. No inclined compression was considered. As confirmed in Figure S1, the intrinsic period of self-oscillation in this system is 51.1 s. The duration of a single compression was set to 10 s. The interval of externally imposed compressions is hereafter referred to as the cycle length (CL) following the previous experimental research.^24^ First, we present the results for CL values in the range of 46, 50, and 54 s, which are close to the intrinsic period of the gel (51.1 s). Figure 6a represents the temporal variation of the gel length along the left–right direction and the spatially averaged *v* under the external periodic compressions of 46, 50, and 54 s. The dashed lines represent the start and end of each individual compression. In all cases, the reaction oscillation period progressively converged to that of the externally applied periodic compressions and was sustained thereafter. Following the termination of compressions, the self-oscillations returned to its intrinsic period of 51.1 s. These results demonstrate that, under the present conditions, resonance occurs between the self-oscillations of the gel and the external periodic compressions. This is clearly confirmed in Figure 6b, showing the variation in the interval between *v* peaks under external periodic compressions. The term peak number denotes the sequential order of oscillatory peaks in Figure 6a. The reaction period gradually shifted from the intrinsic period (51.1 s) and eventually synchronized with the compression periods (CL = 46, 50, and 54 s), rather than exhibiting immediate resonance upon the onset of compression. External compressions are initially applied during the late contraction stage. This causes a gradual delay in the reaction phase, resulting in a slight increase in the oscillation period. For CL = 54 s, the oscillation period gradually increases until synchronization with the external compression interval is achieved. In contrast, when CL is shorter than the intrinsic period, the timing of the external compression gradually shifts to earlier phases of the oscillation cycle and eventually occurs during the late swelling stage. In this regime, the reaction phase advances rather than delays, causing the oscillation period to decrease progressively until synchronization with the external compression interval is reached. This distinction is suggested to underlie the difference in the time required for the gel oscillation period to match the CL.

**Figure 6:**
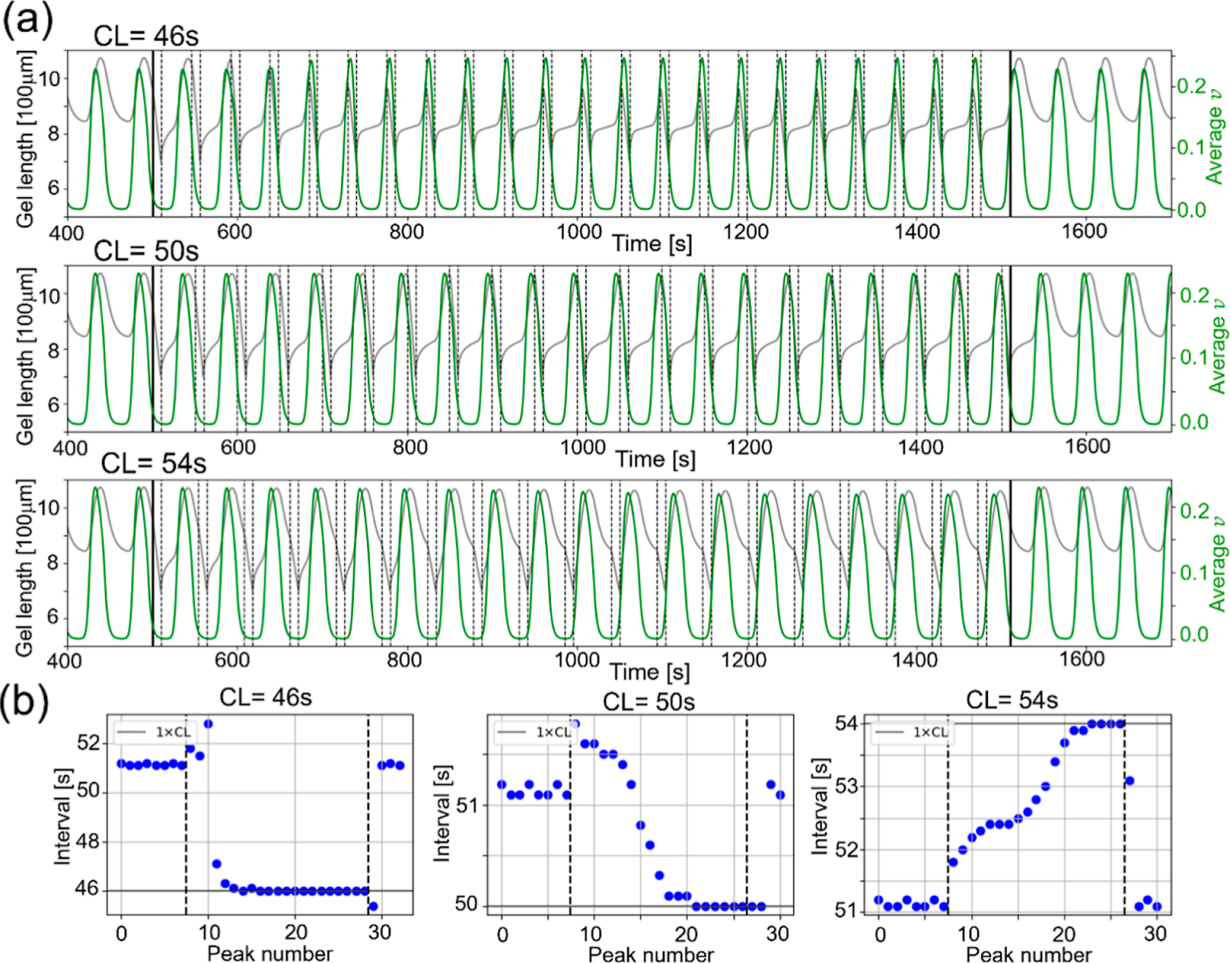
(a) The temporal variation of the gel length along the left–right direction (gray curve) and the spatially averaged *v* (green curve) under the external periodic compressions of 46, 50, and 54 s. The solid lines indicate the start and end of the overall compressions, whereas the dashed lines indicate the start and end of each 10 s compression. (b) Variation in the interval between *v* peaks during oscillation. under the external periodic compressions of 46, 50, and 54 s. Peak number represents the sequential order of oscillatory peaks in the time-series data shown in (a). The dashed lines indicate the onset and termination of external periodic compressions.

Figure 7 shows the results of analogous simulations conducted for external compression periods corresponding to approximately half of the intrinsic period of the gel (25 s), as well as for periods deviating from simple multiple or fractional values (30 and 39 s). When CL was set to 25 s, the externally imposed periodic compressions gave rise to oscillations whose period corresponded to twice the compression period. In a similar manner, Figures S7 and S8 in the Supporting Information show the results for various compression periods corresponding to approximately one-half and one-third of the intrinsic period. Consistent with the case of CL = 25 s, the gel exhibited oscillations whose periods corresponded to two or three times the compression period, thereby confirming resonance at the second and third harmonics of the external oscillations. In contrast, when the external oscillation period deviated from simple fractional values such as one-half, the reaction period did not converge to a constant value and exhibited unstable behavior. When CL was set to 30 s, the interval between peaks exhibited a periodic fluctuation, averaging approximately 60 s, which corresponds to twice the CL, with alternating values of 50 and 70 s. Analysis of the temporal profile of *v* indicated that reactions characterized by two maxima and those characterized by a single peak emerged in an almost alternating manner. When CL was set to 39 s, the peak intervals displayed a periodic variation, yet their average did not align with any integer multiple of CL. The intervals oscillated between the single CL period (39 s) and twice CL (78 s), indicating a repeated oscillatory pattern of roughly one-and-a-half times the CL.

**Figure 7:**
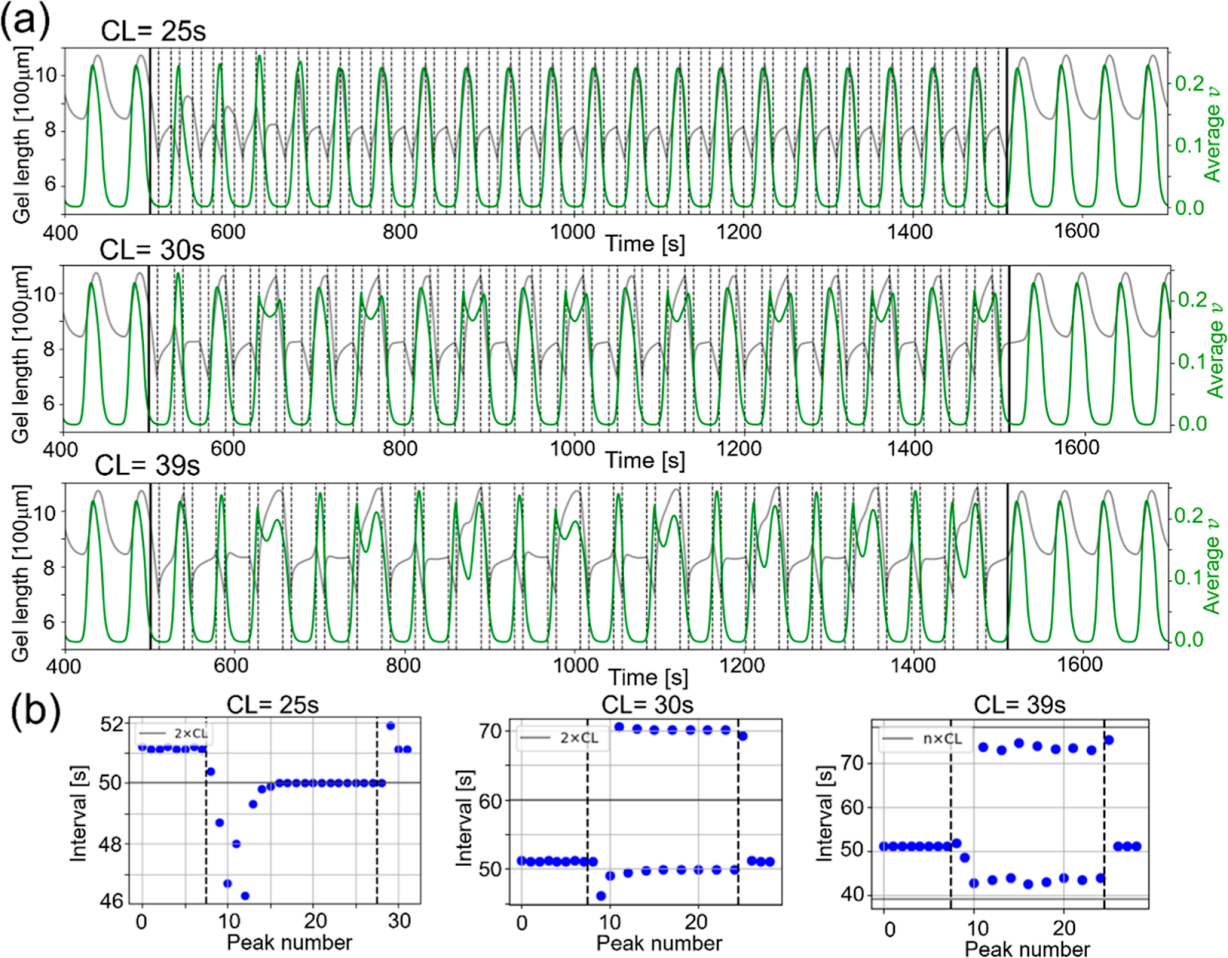
(a) The temporal variation of the gel length along the left–right direction (gray curve) and the spatially averaged *v* (green curve) under the external periodic compressions of 25, 30, and 39 s, which deviate from simple fractional values of the intrinsic period of the gel. The solid lines indicate the start and end of the overall compressions, whereas the dashed lines indicate the start and end of each 10 s compression. (b) Variation in the interval between *v* peaks during oscillation. under the external periodic compressions of 46, 50, and 54 s. Peak number represents the sequential order of oscillatory peaks in the time-series data shown in (a). The dashed lines indicate the onset and termination of external periodic compressions.

Finally, to enable a more detailed discussion, the temporal variation of *v* was subjected to fast Fourier transform (FFT) analysis for further characterization. Figure 8 presents the FFT spectra of the temporal variation of the averaged *v* shown in Figures 6a and 7a. It should be noted that the FFT analysis was performed on the data corresponding to the period during which external compression was applied (from 500 to 1500 s). For CL values of 46, 50, and 54 s, which are close to the intrinsic period of the gel (51.1 s), peaks were observed at the intrinsic period and its harmonics. In the case of CL = 54 s, the frequency is slightly shifted to a lower value. This shift can be attributed to the slow transient behavior before the system reaches steady oscillations with a constant period, as observed in Figure 6b. In the case of CL = 25 s, corresponding to approximately half of the intrinsic period of the gel, not only harmonics on the shorter-period side but also oscillations with a period close to twice the intrinsic period of the gel were observed on the longer-period side. For CL values of 30 and 39 s, which deviate from simple multiples or fractions of the intrinsic period, peaks appeared at CL and its harmonic periods, but several additional peaks were observed at other frequencies. Since it is difficult to determine the presence of harmonics solely from Figures 6 and 7, the confirmation of their existence by FFT analysis is highly significant.

**Figure 8:**
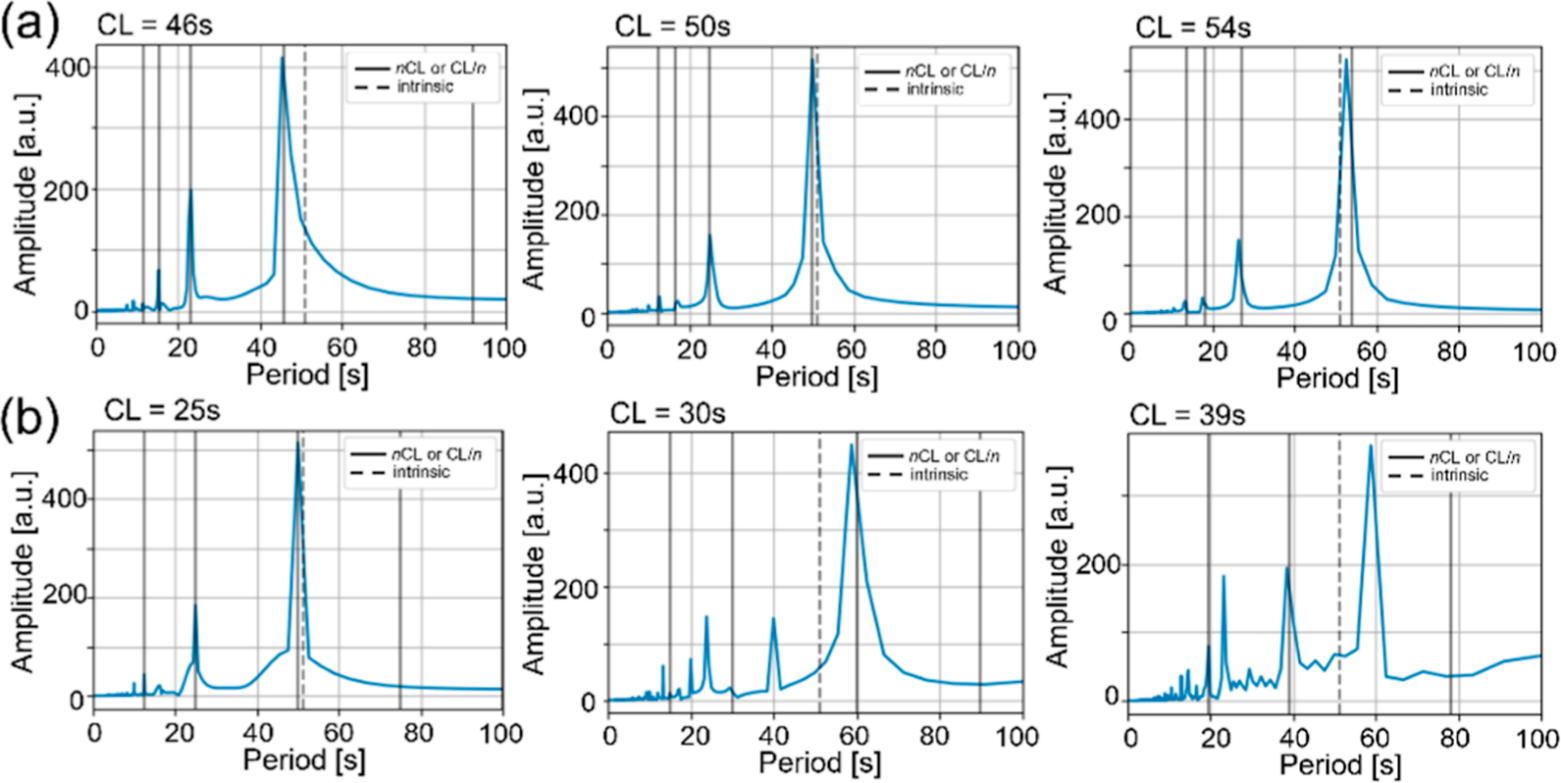
Fast Fourier transform (FFT) spectra of the temporal variation of the averaged *v* under external periodic compressions of (a) 46, 50, and 54 s, as shown in Figure 6, and (b) 25, 30, and 39 s, as shown in Figure 7.

To summarize this section, when external periodic oscillations with frequencies close to the intrinsic period or its harmonics were applied, the gel’s reaction was observed to resonate with the applied oscillations. In contrast, for frequencies farther from these values, quasi-periodic oscillations occurred, which can be attributed to the superposition of waves corresponding to integer multiples or submultiples of the intrinsic period and the compression period. This finding accounts for the results presented in the previous section, in which the gel, having entered a fibrillated state, was able to regain self-oscillations through multiple compressions applied at appropriate intervals. These results demonstrate that, in the self-oscillating gel, mechanical compression can not only induce a fibrillated state but also suppress it under specific conditions. A limitation of the present model is that the oscillation returns to its intrinsic period after the external compression is removed. To investigate the persistence of compression-induced effects, damping mechanisms would need to be explicitly incorporated into the model, which remains an important topic for future study.

## 4 Conclusions

In this study, we numerically investigated the dynamic response of self-oscillating gels to external mechanical forces, with a particular focus on fibrillation control and resonance phenomena. Using a simplified two-dimensional model that couples the BZ reaction with gel volume changes, we successfully reproduced the intrinsic self-oscillations of the gel. A key finding is that inclined compression applied during the early swelling phase can artificially induce fibrillation, which is characterized by stable spiral waves and suppressed swelling and contraction. Furthermore, our simulations revealed that this induced fibrillation could be effectively suppressed, restoring the gel to its self-oscillating state through multiple external compressions. This suppression was most efficient when the compressions were timed at intervals corresponding to integer ratios of the gel’s intrinsic oscillation period, clearly demonstrating a resonance phenomenon. We also observed that the gel’s oscillation period could synchronize with external periodic compressions when their frequencies approached the gel’s intrinsic period or its harmonics. These results underscore the significant potential for precisely regulating self-oscillating gels using mechanical stimulation. The observed similarities between gel fibrillation and cardiac fibrillation suggest that the present model provides valuable insights into the mechanisms underlying biological rhythm disorders. These findings further indicate its potential utility as a framework for developing more effective strategies for their control and resuscitation. Overall, this research demonstrates the potential of ALife approaches for elucidating complex biological dynamics that are difficult to study directly in living organisms.

While the present study focused on the internal mechanical response of the gel, the model can be readily extended to account for interactions between swelling–deswelling dynamics and external fields. This would allow the simulation of more complex behaviors, such as gel rotation and locomotion in diverse environments.^23,29^ By providing a flexible and extensible computational platform, the present framework opens new opportunities for exploring emergent dynamics in active soft matter and biologically inspired systems.

## Supplementary Material











## Notes

Movie for the self-oscillation of the rectangle gel (MP4)
Movie for the fibrillation development in the self-oscillating gel induced by external compression (MP4)
Movie for the response of the self-oscillating gel in the fibrillation state to a single external compression, which fails to suppress fibrillation (MP4)
Movie for the response of a self-oscillating gel in the fibrillation state to two external compressions, resulting in the suppression of fibrillation (MP4)
A Mathematical Model of Self-Oscillating Gel (Section S1) ; Validation of Self-Oscillation in a Square Gel (Section S2); Snapshots of self-oscillation in a square gel for validation (Figure S1); Snapshots of various patterns in a square self-oscillating gel (Figure S2); Detailed snapshots showing the manifestation of fibrillation (Figures S3, S4 and S5); the temporal variation of the gel length during applying additional compression (Figures S6, S7 and S8) (PDF)
