# Supplementary material for: Artificial Life Approach for Controlling Fibrillation in Self-Oscillating Gels
Source: ACS Omega. 2026 Jul 24;11(31):46419–28. doi: 10.1021/acsomega.6c03790 (PMC13470717; doi:10.1021/acsomega.6c03790)
Supplement: Supplementary file 1 [file ao6c03790_si_005.pdf]

Artificial Life Approach for Controlling Fibrillation in Self-Oscillating Gels

Shun Oi, Ryuhei Sato, Ryo Yoshida, Yasushi Shibuta*

Department of Materials Engineering, The University of Tokyo, 7-3-1 Hongo, Bunkyo-ku, Tokyo 113-8656, Japan

*Corresponding author. Email: shibuta@material.t.u-tokyo.ac.jp (YS)

Section S1 A Mathematical Model of Self-Oscillating Gel

The mathematical model of the self-oscillating gel employed in this study is essentially based on the work of Yashin et al.[r1] and is formulated as a simplified two-dimensional model to facilitate implementation. In overview, reproducing the behavior of self-oscillating gels requires coupling a model that describes the internal BZ reaction with a model that accounts for the structural changes of the gel. In the swelled gel, it is assumed that the polymer network is bound to the metal ion catalyst and occupies a volume fraction ϕ. The remaining BZ reactants are dissolved in the solvent, which occupies the remaining volume fraction $(1-\phi)$. The polymer volume fraction ϕ for each element is calculated from its volume V, initial polymer fraction ϕ_0, and initial volume V_0 as follows:

$$\phi = \frac{\phi_0 V_0}{V} \tag{S1}$$

where V of each element can be determined from the lattice point coordinates of the simulation system.

A modified Keener-Tyson model is employed to describe the BZ reaction within the gel. The dimensionless concentrations of the activator u (proportional to $HBrO_2$ concentration) and the inhibitor v (proportional to Ce^{4+} concentration) are described by the following equations:

$$\frac{\partial u}{\partial t} = F(u, v, \phi) \tag{S2}$$

$$\frac{\partial v}{\partial t} = \varepsilon G(u, v, \phi) \tag{S3}$$

Here, the reaction terms $F(u, v, \phi)$ and $G(u, v, \phi)$ are given by:

$$F(u, v, \phi) = (1-\phi)^2 u - u^2 - (1-\phi) f v \frac{u - q(1-\phi)^2}{u + q(1-\phi)^2} \tag{S4}$$

$$G(u, v, \phi) = (1-\phi)^2 u - (1-\phi) v \tag{S5}$$

where ε is the ratio of reaction rates, and q and f are reaction parameters. These equations describe the dynamic concentration changes of the BZ reaction within the gel. To describe the dynamics of the reactive gel, a two-fluid model is adopted. This model assumes that the velocity of the polymer $\boldsymbol{v}^{(p)}$ and the velocity of the solvent $\boldsymbol{v}^{(s)}$ contribute to the overall velocity $\boldsymbol{v}$. These velocities are related by the following equation:

$$\boldsymbol{v}^{(p)}(1-\phi)+\boldsymbol{v}^{(s)}\phi=\boldsymbol{v} \qquad (S6)$$

The system is assumed to be incompressible, meaning $\nabla\cdot\boldsymbol{v}=0$. To incorporate transport processes into the BZ reaction model, corresponding diffusion and advection terms are added to the reaction equations (S2) and (S3):

$$\frac{\partial u}{\partial t}=-\nabla\cdot\boldsymbol{j}(u)-\nabla\cdot\left(u\boldsymbol{v}^{(s)}\right)+F(u,v,\phi) \qquad (S7)$$

$$\frac{\partial v}{\partial t}=-\nabla\cdot\left(v\boldsymbol{v}^{(p)}\right)+\varepsilon G(u,v,\phi) \qquad (S8)$$

Here, $\boldsymbol{j}(u)$ represents the diffusion flux, which is expressed by the following equation:

$$\boldsymbol{j}(u)=-D_u(1-\phi)\nabla\left(u(1-\phi)^{-1}\right) \qquad (S9)$$

Furthermore, the continuity equation for the polymer volume fraction is expressed as:

$$\frac{\partial \phi}{\partial t}=-\nabla\cdot\left(\phi\boldsymbol{v}^{(p)}\right) \qquad (S10)$$

Assuming the overall velocity of the system $\boldsymbol{v}=0$, the solvent velocity $\boldsymbol{v}^{(s)}$ is derived from the polymer velocity $\boldsymbol{v}^{(p)}$ as:

$$\boldsymbol{v}^{(s)}=-\frac{\phi}{1-\phi}\boldsymbol{v}^{(p)} \qquad (S11)$$

A gel lattice spring model is adopted to describe the flexibility of the gel following the work of Yashin et al.[r1] This model discretizes the object into lattice points, with each point connected by springs. The key differences from a conventional lattice spring model are the introduction of

osmotic pressure and the assumption of overdamped oscillation for the movement of lattice points. The osmotic pressure π_{osm} is defined considering the Flory-Huggins theory and the change in hydrophilicity due to the oxidation of metal ions within the gel. It is calculated by the following equation:

$$\pi_{osm} = -[\phi - \ln(1-\phi) + \chi(\phi)\phi^2] + \chi^* v\phi \qquad (S12)$$

Here, $\chi(\phi)$ is the polymer-solvent interaction parameter, which is expressed as:

$$\chi(\phi) = \chi_0(T) + \chi_1\phi \qquad (S13)$$

The force $\boldsymbol{F}_{k,l}$ acting on each lattice point (k, l) is determined by the elastic forces between neighboring lattice points and the pressure from osmotic forces.

$$\boldsymbol{F}_{k,l} = \frac{K}{3}\sum_{\delta,\delta'=0,\pm1}\left(\boldsymbol{x}_{(k+\delta,l+\delta')} - \boldsymbol{x}_{(k,l)}\right) + \Big(\boldsymbol{e_3} \times \left(\boldsymbol{x}_{(k,l+1)} - \boldsymbol{x}_{(k+1,l)}\right)\pi(k,l) + \boldsymbol{e_3} \times \left(\boldsymbol{x}_{(k-1,l)} - \boldsymbol{x}_{(k,l+1)}\right)\pi(k-1,l) + \boldsymbol{e_3} \times \left(\boldsymbol{x}_{(k,l-1)} - \boldsymbol{x}_{(k-1,l)}\right)\pi(k-1,l-1) + \boldsymbol{e_3} \times \left(\boldsymbol{x}_{(k+1,l)} - \boldsymbol{x}_{(k,l-1)}\right)\pi(k,l-1)\Big) \qquad (S14)$$

where $\boldsymbol{x}_{(k,l)}$ represents the coordinates of lattice point (i, j), K is the spring constant, and $\boldsymbol{e_3}$ is the unit vector perpendicular to the *xy*-plane. The motion of each lattice point $\boldsymbol{m} = (k, l)$ is described as an overdamped oscillation without inertia:

$$\frac{\partial \boldsymbol{x_m}}{\partial t} = M_m \boldsymbol{F}_m \qquad (S15)$$

Here, M_m is called the mobility, indicating that the velocity of the gel lattice point is proportional to the force acting on that part. M_m is calculated by the following equation:

$$M_m = 4\Lambda_0(1-\phi_m)\left(\frac{\phi_m}{\phi_0}\right)^{-\frac{1}{2}} \qquad (S16)$$

where ϕ_m is the average polymer volume fraction of the four elements surrounding lattice point $\boldsymbol{m}$, and Λ_0 is the friction coefficient.

Section S2 Validation of Self-Oscillation in a Square Gel

Here, the self-oscillating gel model was verified to be correctly implemented and capable of reproducing self-oscillation. Figure S1(a) shows snapshots of self-oscillation of the square gel starting from $u = 0.001$, $v = 0.001$ and $\phi = 0.139$, while Figure S1(b) shows the temporal evolution of the gel length along the left–right direction together with the spatially averaged values of u and v over the entire gel. The color of the snapshot indicates the concentration distribution of v, which is applied consistently in the subsequent snapshots. The corresponding dynamics are provided in Supplementary Movie S1. The BZ reaction occurred within the gel, leading to repeated increases and decreases in u and v, which in turn caused periodic oscillations in the gel size. The gel length along the left–right direction was 1070 μm in the swollen state and 840 μm in the contracted state, corresponding to a 1.27-fold increase upon swelling. For the spatially averaged concentrations of each reactant, v ranged from a minimum of 0.00030 to a maximum of 0.228, and u ranged from a minimum of 0.00090 to a maximum of 0.29830. The oscillation period, determined from the interval between successive peaks of v, was 51.1 s. This result confirms the validity of the model and demonstrates its capability to accurately reproduce the intrinsic oscillatory and structural dynamics of the self-oscillating gel through numerical simulation.

Next, to further validate the physical accuracy of the present model, the changes in internal patterns of a two-dimensional square-shaped self-oscillating gel were compared with experimental results.[r2] Here, the grid resolution was set to 20 × 20 to examine the internal patterns in detail, while all other conditions were kept the same as those described above. Figure S2 shows snapshots

of the self-oscillating gel at the early stage of the reaction and after a certain elapsed time. At the early stage, as illustrated in the figure, the reaction first progresses from the four corners of the square, where the effect of diffusion is strong, and subsequently propagates throughout the entire domain. The preferential diffusion from the four corners gives rise to concentration distributions forming a cross or crisscross pattern. A similar behavior was observed in the experimental system;[r2] that is, BZ waves propagated from the corners of the square gel, resulting in a similar cross pattern in the distribution of BZ waves (see Figure 2 in ref. (r2) for details). Furthermore, at later times, the reaction initiates from the center and diffuses outward in concentric circles, resulting in an almost uniform pattern throughout the system. A similar pattern of BZ waves was also observed in the experimental system (see Figure 3 in ref. (r2) for details). Comparison with these experimental results confirms that our simulations accurately reproduce pattern formation both at the early stage of the reaction and during the stable phase.

(r1) Yashin, V. V.; Balazs, A. C. Theoretical and computational modeling of self-oscillating polymer gels. *J. Chem. Phys.* **2007**, *126*, 124707.

(r2) Chen, I. C.; Kuksenok, O.; Yashin, V.V.; Moslin, R.M.; Balazs, A.C.; Van Vliet, K.J. Shape- and size-dependent patterns in self-oscillating polymer gels. *Soft Matter.* **2011**, *7*, 3141–3146.

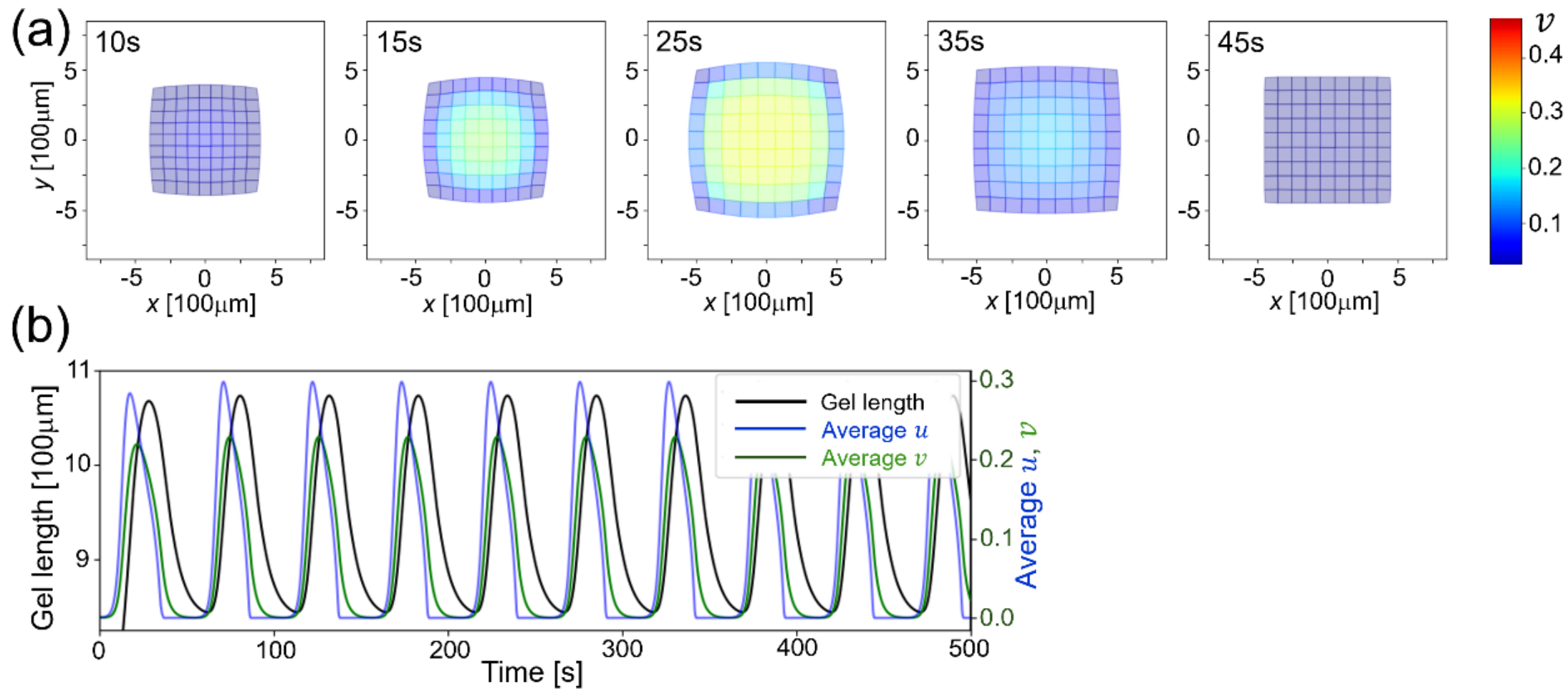

Figure S1 (a) Snapshots of self-oscillation in a square gel with initial concentrations, $u = 0.001$, $v = 0.001$ and $\phi = 0.139$. Colors indicate the concentration distribution of v. (b) Temporal evolution of the gel length along the left–right direction (black curve) together with the spatially averaged values of u (blue curve) and v (green curve) over the entire gel. The intrinsic period of the gel is estimated to be 51.1 s from the periodic oscillation observed in the graph.

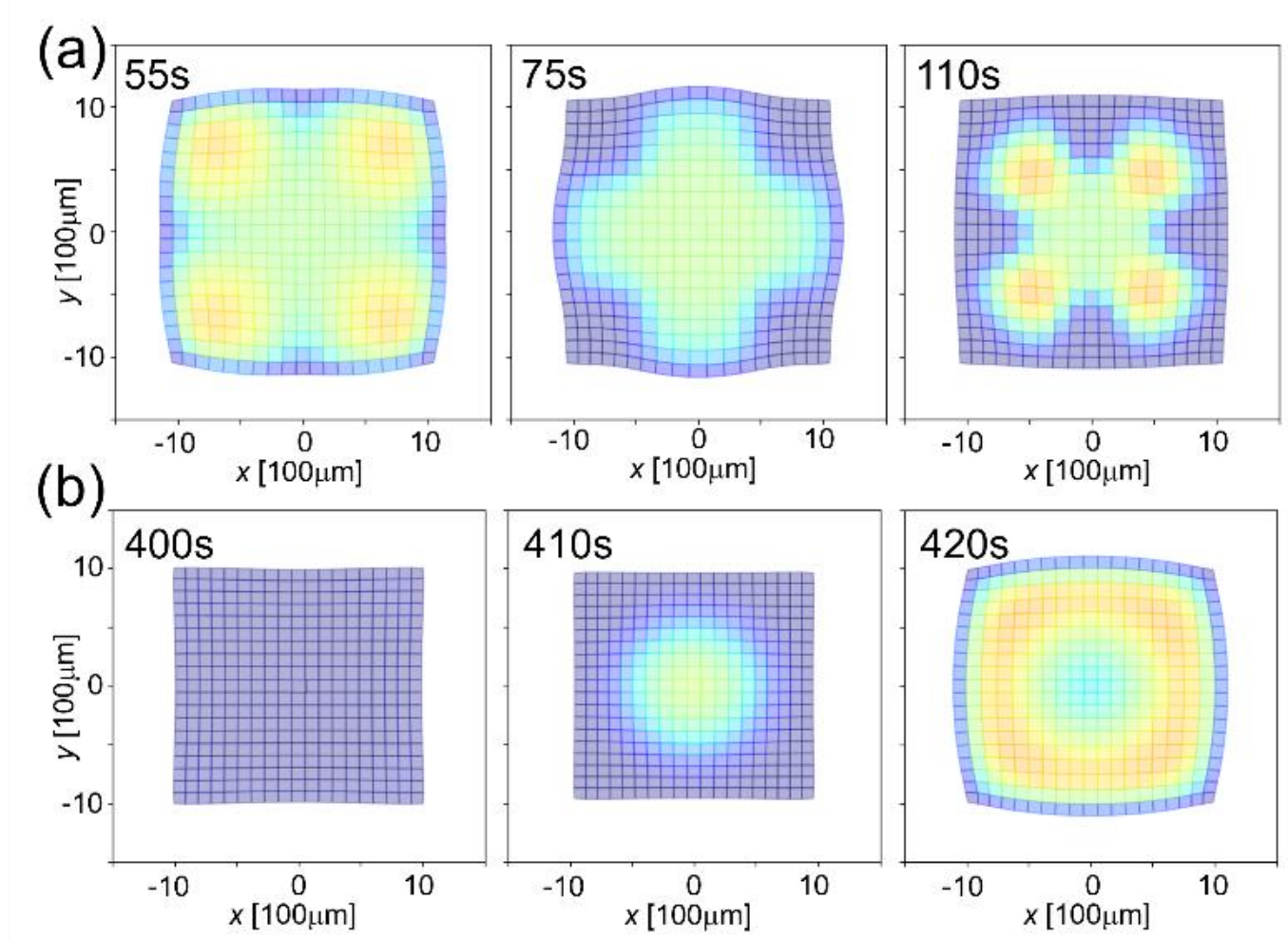

Figure S2 Snapshots of various patterns in a square self-oscillating gel: (a) at the early stage of the reaction and (b) after a certain elapsed time, for comparison with experimental results. Colors indicate the concentration distribution of v.

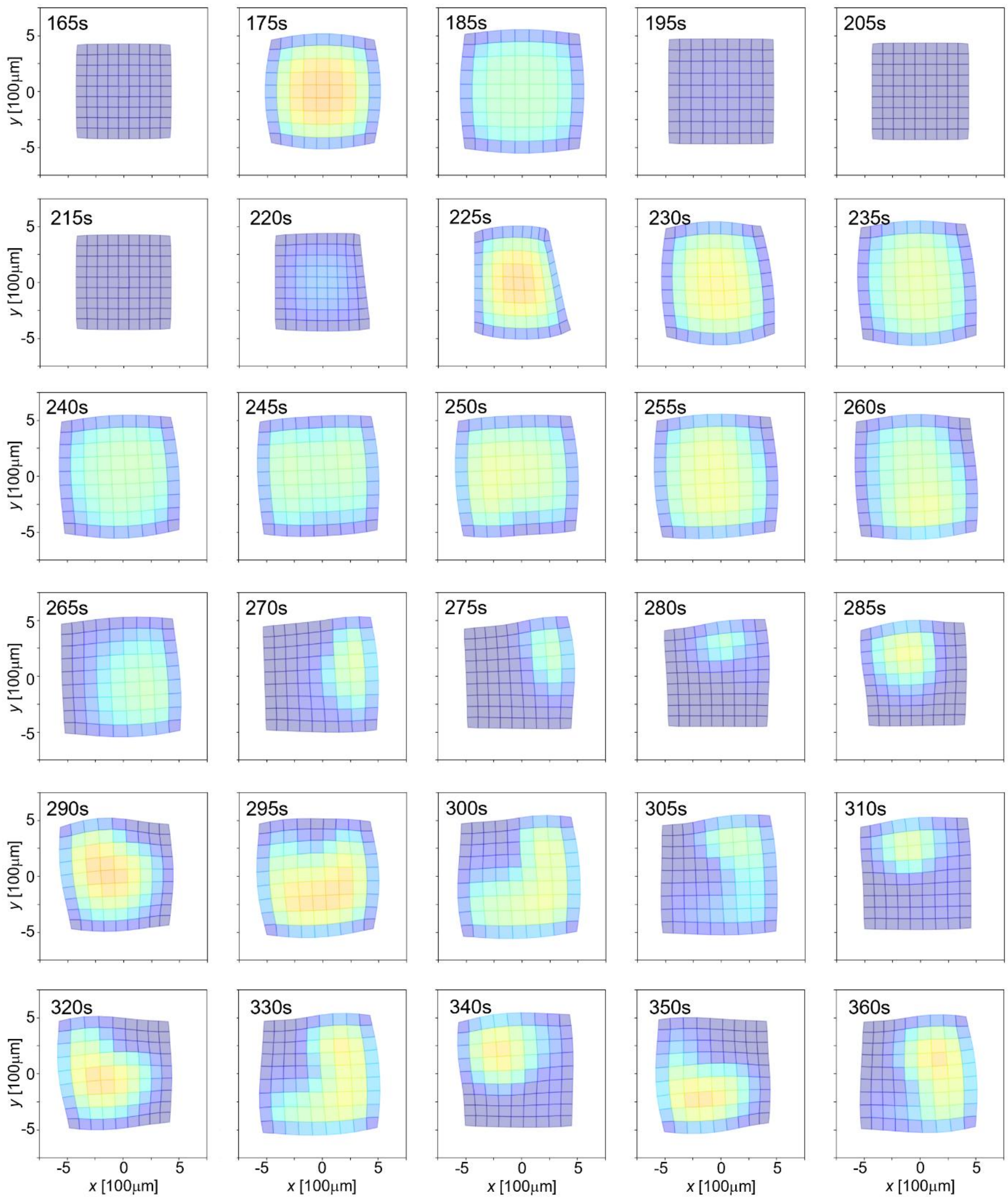

Figure S3 Detailed snapshots from 165 to 360 s showing the manifestation of fibrillation when the upper end was compressed down to 60% and the lower end down to 80% of the swollen-state length (1070 μm). Colors indicate the concentration distribution of v.

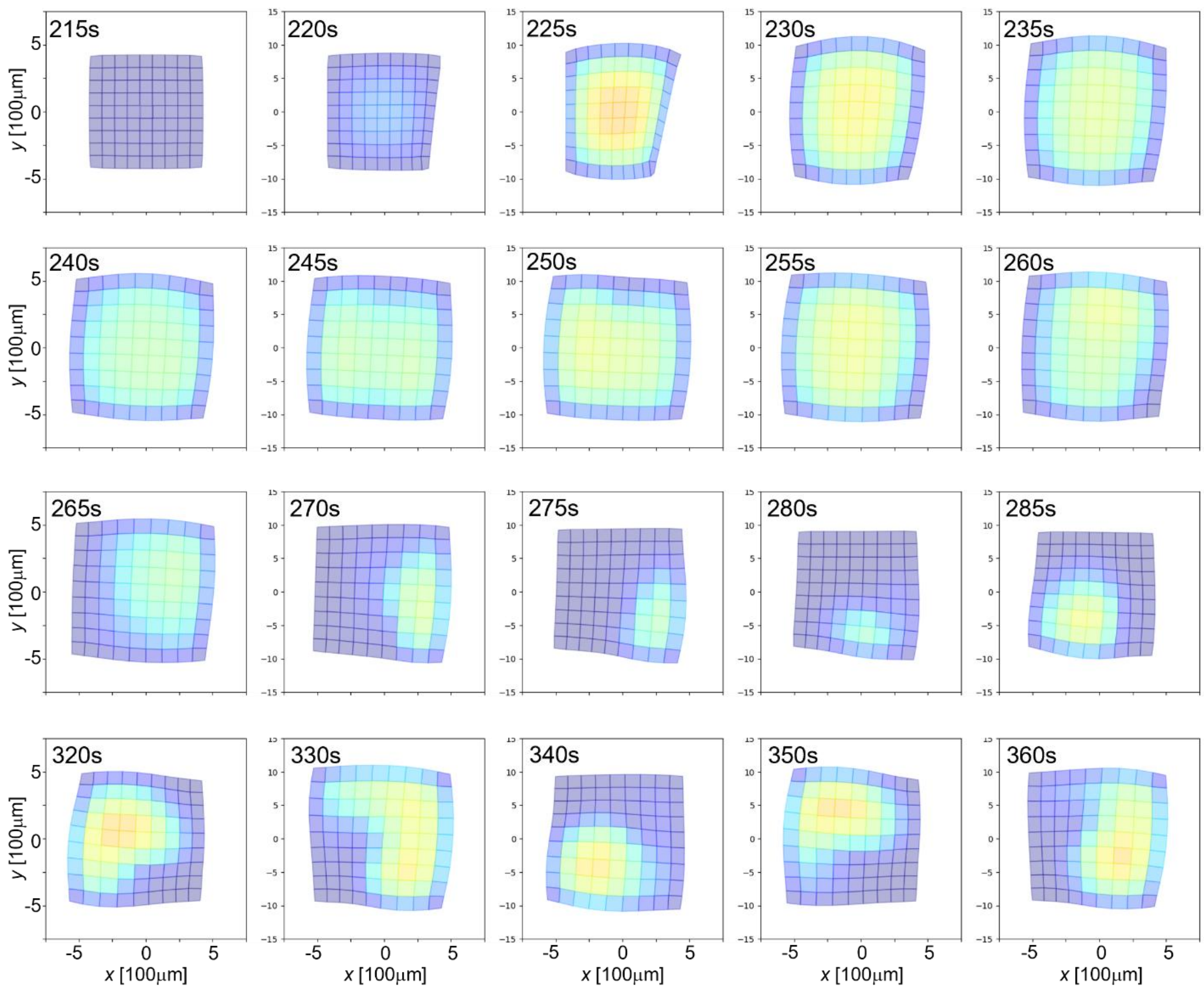

Figure S4 Detailed snapshots from 215 to 360 s showing the manifestation of fibrillation when the upper end was compressed down to 80% and the lower end down to 60% of the swollen-state length (1070 μm). Colors indicate the concentration distribution of v.

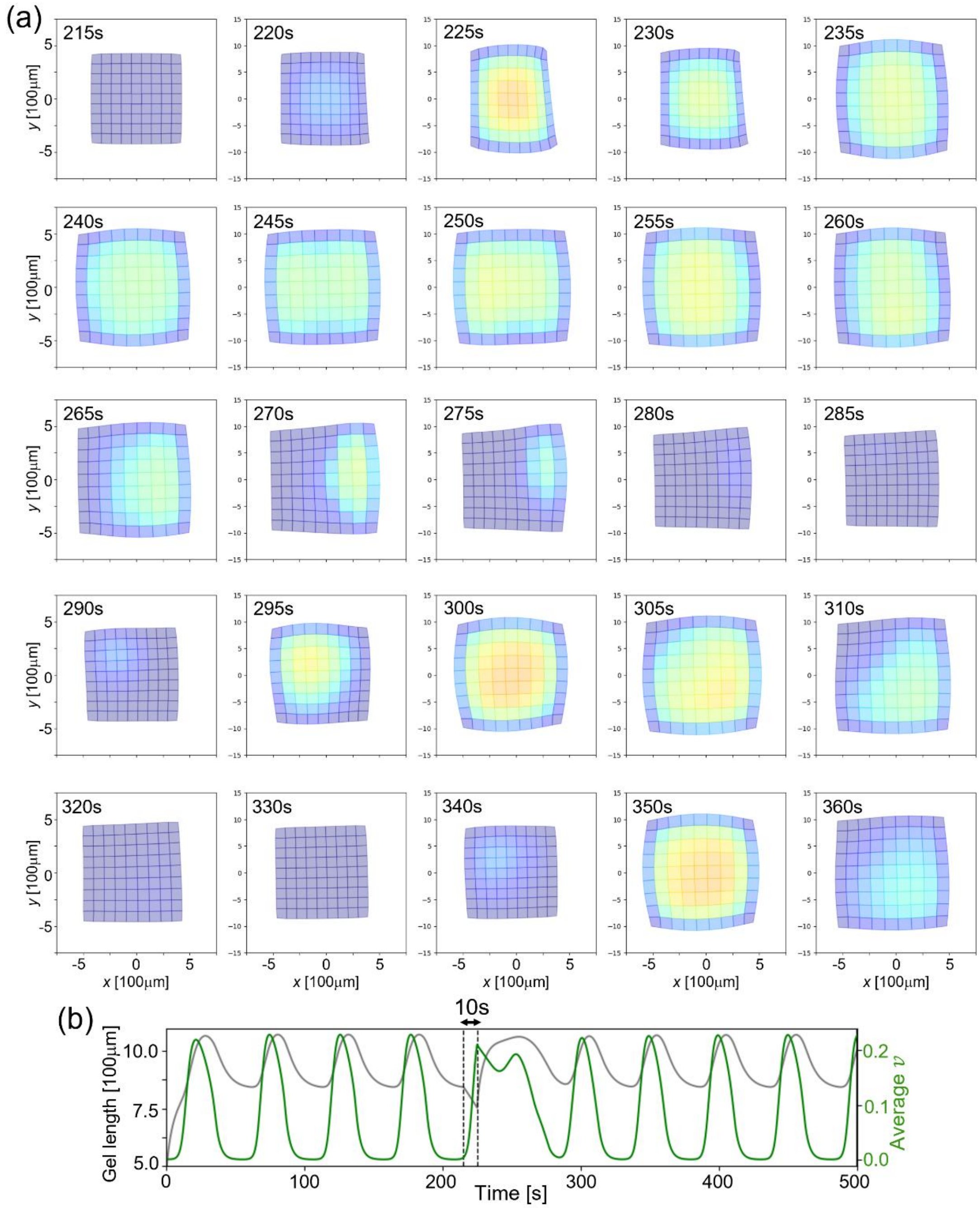

Figure S5 (a) Detailed snapshots from 215 to 360 s showing the manifestation of fibrillation when the upper end was compressed down to 70% and the lower end down to 75% of the swollen-state length (1070 μm). Colors indicate the concentration distribution of v. (b) The temporal variation of the gel length in the left–right direction (gray curve) and the spatially averaged v (green curve). The interval between the dotted lines corresponds to the 10 s period during which the gel is subjected to compression.

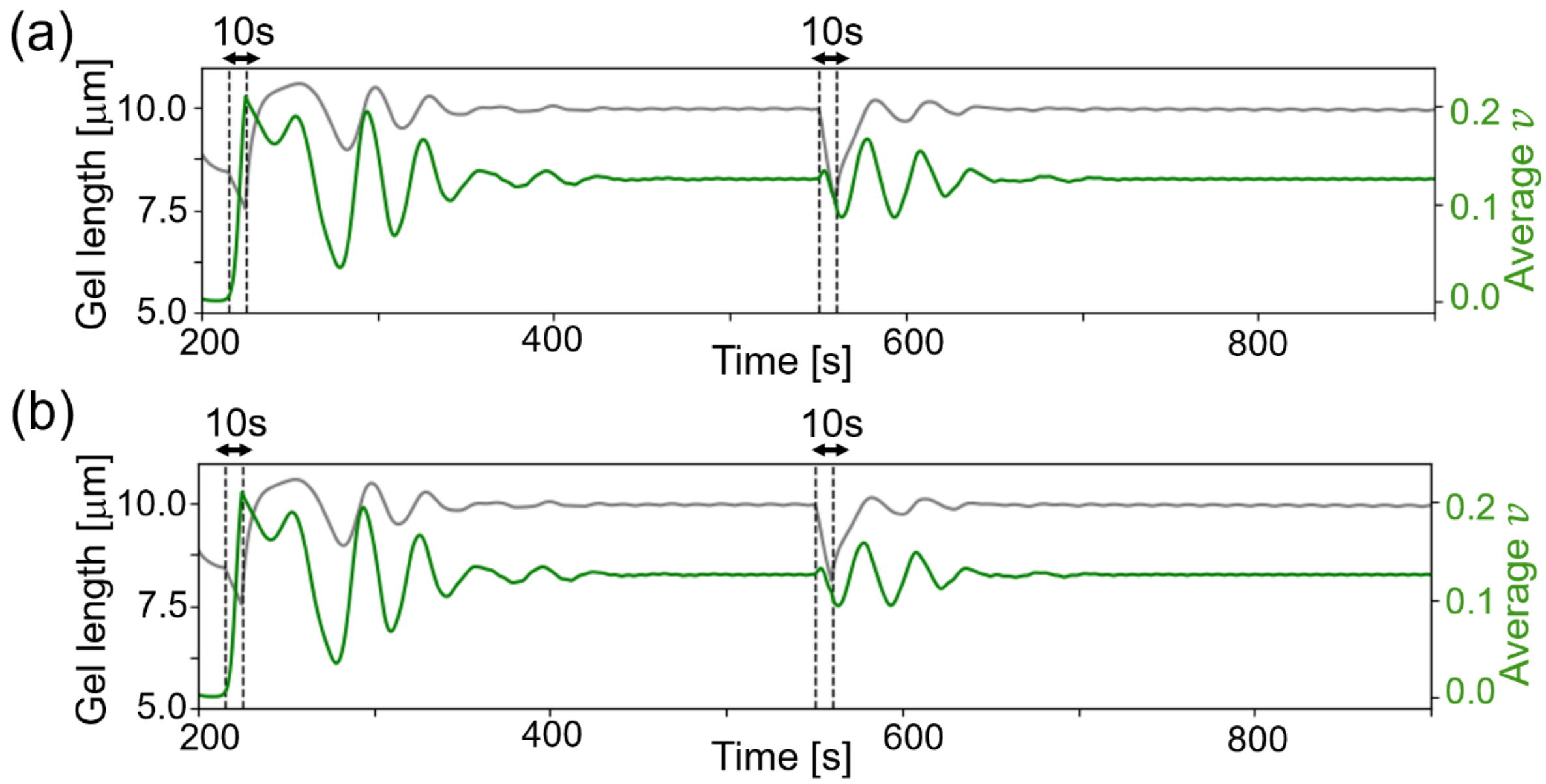

Figure S6 The temporal variation of the gel length along the left–right direction (gray curve) and the spatially averaged v (green curve) during applying a single additional compression down to (a) 75% and (b) 80% of the swollen-state length at 550 s. The interval between the dotted lines corresponds to the 10 s period during which the gel is subjected to compression.

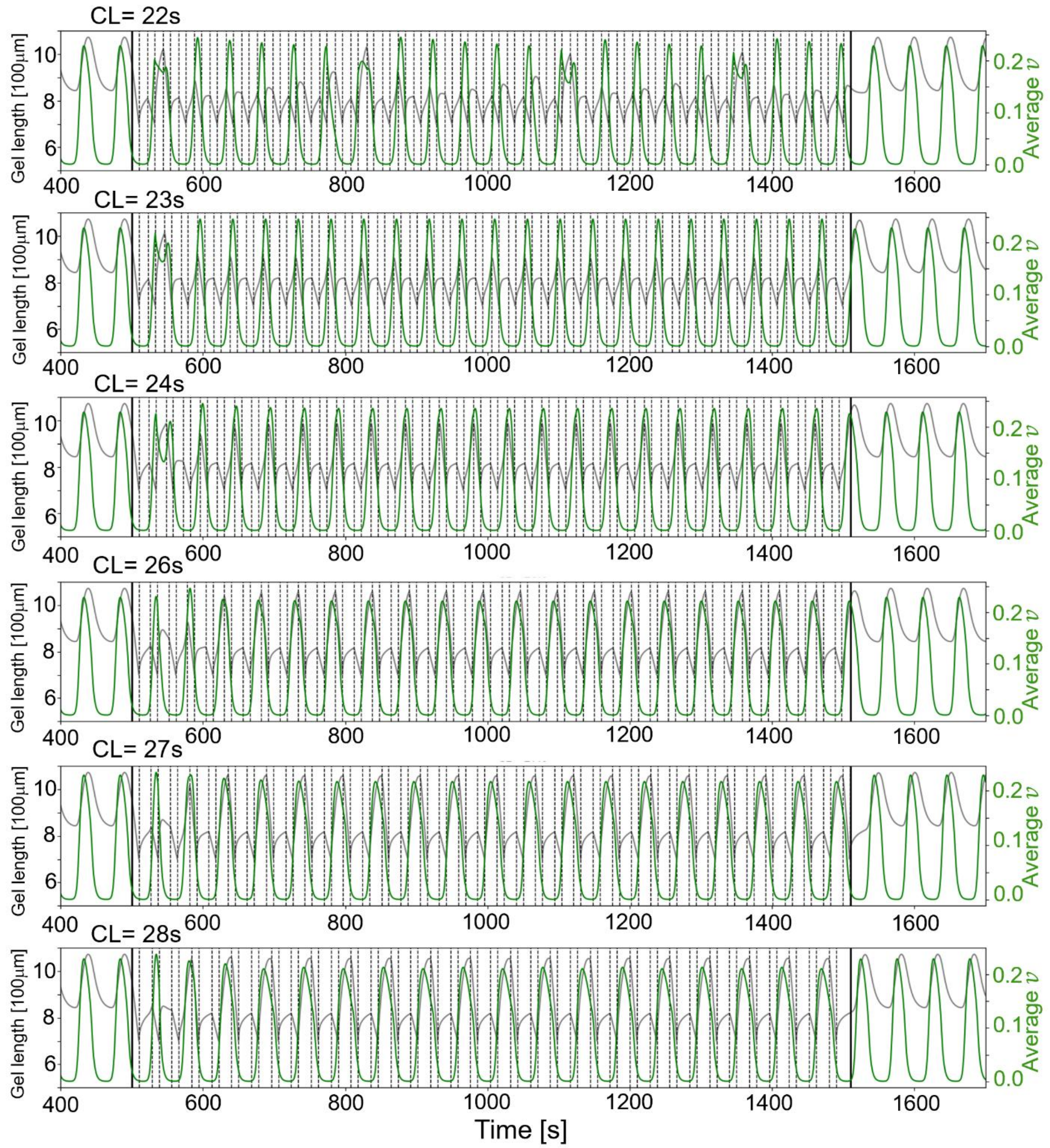

Figure S7 The temporal variation of the gel length along the left–right direction (gray curve) and the spatially averaged v (green curve) under the external periodic compression of approximately one-half of the intrinsic period. The solid lines indicate the start and end of the overall compression, whereas the dashed lines indicate the start and end of each 10 s compression.

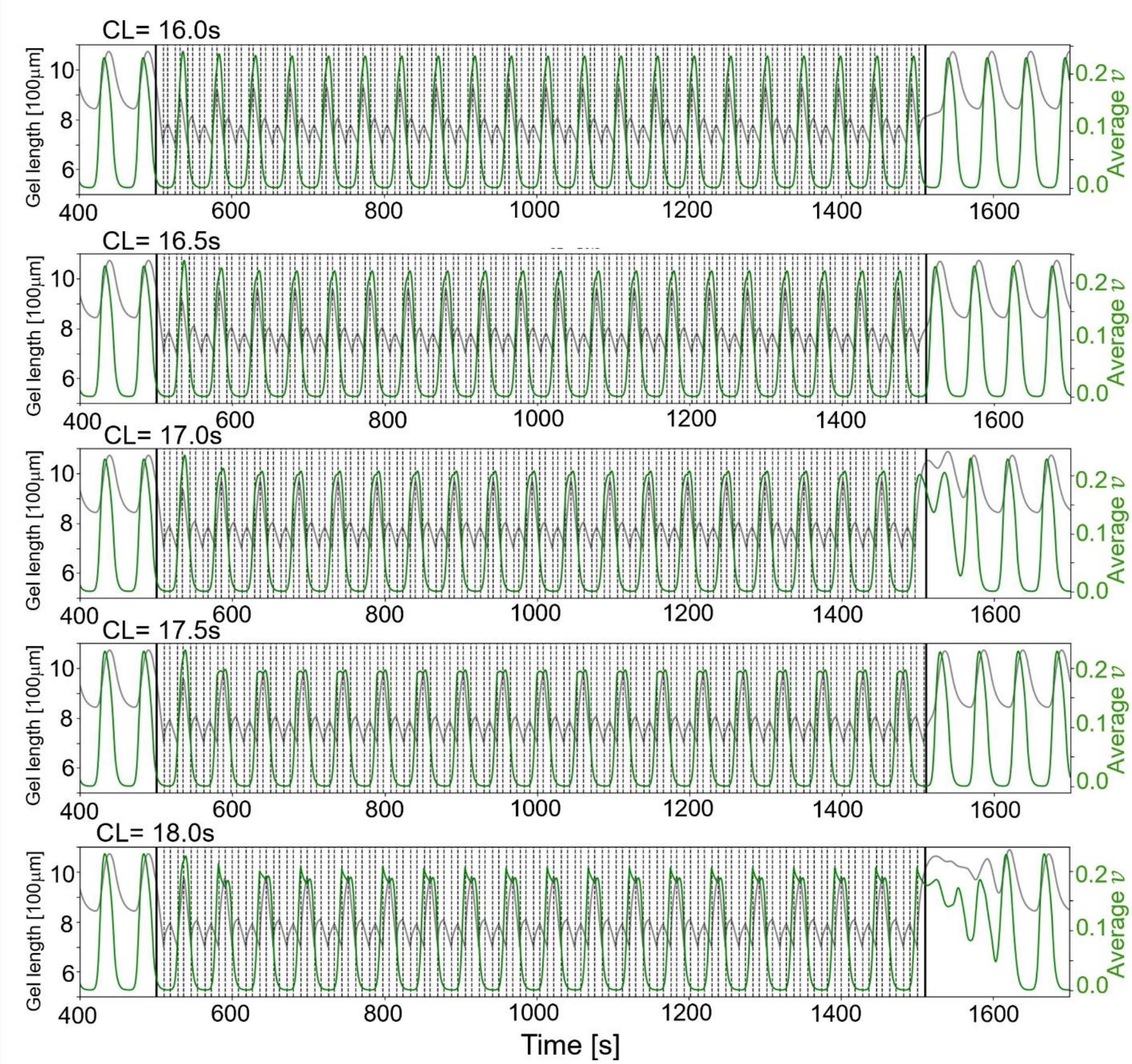

Figure S8 The temporal variation of the gel length along the left–right direction (gray curve) and the spatially averaged v (green curve) under the external periodic compression of approximately one-third of the intrinsic period. The solid lines indicate the start and end of the overall compression, whereas the dashed lines indicate the start and end of each 10 s compression.

Movie S1.

Movie for the self-oscillation of the rectangle gel starting from $u = 0.001$, $v = 0.001$ and $\phi = 0.139$.

Movie S2.

Movie for the fibrillation development in the self-oscillating gel induced by external compression.

Movie S3.

Movie for the response of the self-oscillating gel in the fibrillation state to a single external compression, which fails to suppress fibrillation.

Movie S4.

Movie for the response of a self-oscillating gel in the fibrillation state to two external compressions, resulting in the suppression of fibrillation.
